# Effect of *Phaseolus Vulgaris* on Urinary Biochemical Parameters among Patients with Kidney Stones in Saudi Arabia

**DOI:** 10.3390/nu12113346

**Published:** 2020-10-30

**Authors:** Sahbanathul Missiriya Jalal, Abdulrahman Abdulhadi Alsultan, Hala Hazam Alotaibi, Ester Mary, Abeer Abbas Ibrahim Alabdullatif

**Affiliations:** 1 Department of Nursing, College of Applied Medical Sciences, King Faisal University, Al-Ahsa 31982, Saudi Arabia; ab3eer@hotmail.com; 2 Department of Biomedical Sciences, College of Medicine, King Faisal University, Al-Ahsa 31982, Saudi Arabia; aalsultan@kfu.ed.sa; 3 College of Applied Medical Sciences, King Faisal University, Al-Ahsa 31982, Saudi Arabia; 4 Department of Food Sciences and Nutrition, College of Agricultural and Food Sciences, King Faisal University, Al-Ahsa 31982, Saudi Arabia; hhalotaibi@kfu.edu.sa; 5 Department of Pediatrics, King Khalid Hospital, Najran 66262, Saudi Arabia; epappiya@moh.gov.sa

**Keywords:** *Phaseolus Vulgaris*, urinary biochemical parameters, kidney stones, renal calculi

## Abstract

The study purpose was to investigate the effect of *Phaseolus Vulgaris* (PV) on urinary biochemical parameters among patients with kidney stones. We conducted a randomized controlled study among 60 patients with kidney stones (size < 10 mm) in the nephrology unit of both government and private hospitals, Al-Ahsa. Urinary volume, calcium, magnesium, potassium, oxalate, uric acid, and power of hydrogen (pH) were assessed before and after the intervention of giving 250 g of PV consumption as an extract thrice weekly (2.2 L to 2.5 L per week) for 6 weeks, which was compared with control. A ‘t’ test was used with the significance at 5%. Mean score of age was 44.5 ± 10.16 in PV group and 43.73 ± 9.79 in control. Four (13.3%) and two (6.7%) had family history of kidney stones. Body mass Index (BMI) mean was 26.44 ± 2.7 and 26.36 ± 2.65 in pre and post-test, respectively, which were significant (*p* = 0.01017). There were significant changes (*p* = 0.000) in urine volume from 1962 ± 152.8 to 2005 ± 148.8, calcium 205.4 ± 11.99 to 198.4 ± 12.52, potassium 44.07 ± 3.66 to 52.15 ± 4.37, oxalate 37.12 ± 5.38 to 33.02 ± 5.71, and uric acid 6.88 ± 0.7 to 6.31 ± 0.58. In conclusion, PV is effective management for the patients with kidney stones as it increases the urinary volume and enhances the elimination of small kidney stones.

## 1. Introduction

Kidney stones or renal calculi are deposition of minerals as a crystal concretion of organic components, mostly found in the kidney calyces and pelvis [1]. This is a common and major human health issue globally and 12% of the population are affected. The prevalence and incidence of renal calculi is reported to be increasing across the world [2]. In different varieties of kidney stones, calcium oxalate (CaOx) stones are the most common and they formed at Randall’s plaque on the renal papillary surfaces. The CaOx stones are mixed with calcium phosphate and those are the most prevalent (80%) across the world. The other types of kidney stones such as uric acid, struvite, and cystine are also prevalent at 9%, 10%, and 1%, respectively [3].

The incidence report obtained from various countries demonstrated that among 100,000 individuals, 114–720 people were affected with kidney stones. The number of cases is revealed by prevalence rates of 1.7%–14.8% worldwide [4,5]. Gender wise statistics showed that kidney stones were prevalent among approximately 13% of men and 7% of women [6].

The renal calculi formation and prevalence rate depended upon the geographical variations, based on the climate, with a high occurrence evidenced in hot places [7]. Some of the predisposing factors included inadequate fluid intake, concentrated and decreased urine output, and excessive perspiration, which enhances stone formation by crystallization [8].

Studies showed that the occurrence of kidney stones was higher during the hot season. Saudi Arabia is one of the hot climate countries. During the summer season, the temperatures in the eastern province of Saudi Arabia will rise to 50 °C at its extreme level [7]. Saudis are 2.5 times more likely to develop urolithiasis in hot regions. Furthermore, 50% recurrence rate is experienced by people with kidney stones [4]. Dietary modifications, lifestyle changes, and the use of specific medications are necessary to reduce the recurrence of renal calculi [9].

Management of this problem depends on the size and location of the stones. There are complimentary therapies and conventional treatments available for renal calculi particularly in non-severe conditions [10]. *Phaseolus Vulgaris* (PV) is a bean vegetable from the family of pea, which is used to reduce kidney stone formation. These are a great source of minerals and B vitamins that help in cleaning the kidneys and enhancing urinary tract functions. PV provides a rich variety of phytochemicals, such as protein, amino acids, dietary fibers, folate, iron, potassium, magnesium, complex carbohydrates, oligosaccharides, phenols, saponins, flavonoids, alkaloids, and tannins, while containing little or no total fat, trans-fat, sodium, or cholesterol, and has potential health benefits [11,12] The seeds of PV have been claimed to possess the diuretic activity and, therefore, PV seeds are used in pregnant women to reduce the water retention of the body [13].

There are animal studies that proved the effect of PV on kidney stones [14]. Additionally, some clinical trials revealed that phytotherapeutic agents [15] could be used as either an alternative or an adjunctive therapy in the management of urolithiasis. However, human clinical studies on these issues are lacking. Therefore, in the present study, we aimed to investigate the effect of PV on urinary biochemical parameters among patients with kidney stones.

## 2. Materials and Methods

### 2.1. Study Design

As a randomized controlled trial, this study was conducted to determine the effect of PV on urinary biochemical values, among patients with kidney stones in the nephrology units of governmental and non-governmental hospitals in Al-Ahsa, Saudi Arabia. The study protocol was approved by the King Fahad Hospital, Hofuf, Institutional Review Board (H-05-HS-065), and it was registered (KFHH RCA 26-29-2020) in the Directorate of Health Affairs in Al-Ahsa. The objective and the procedures performed in the study were fully understood by the participants, and all of them provided informed consent for inclusion in the study. This study was conducted in accordance with the Declaration of Helsinki and followed the ethical principles.

### 2.2. Participants

The inclusion criteria were patients aged from 20 to 60 years, including both male and female genders residing in the Al-Ahsa region with renal calculi < 10 mm in size based on ultrasonography findings. Patients with a serum creatinine level > 2.0 mg/dL [16], urinary tract infection, uncontrolled diabetes, chronic liver disease, renal failure, cancer, Corona Virus Disease-19 (COVID-19), serious illness, complicated diseases, pregnant and lactating women, or known allergies to PV were in exclusion criteria because these factors were associated with the exacerbation of symptoms or serious damage clinically.

### 2.3. Sample Size

The sample size was estimated considering the mean and standard deviation of a similar, previous study with an α error of 0.05 and β error of 0.20. Accounting for 10% dropouts, the total sample size was calculated as 29.5 in each group, which was rounded off to 30 in each group. Hence, a total of 60 patients with kidney stones were selected to determine the effect of PV on the urinary biochemical parameters.

### 2.4. Experimental Methods

**Figure 1:**
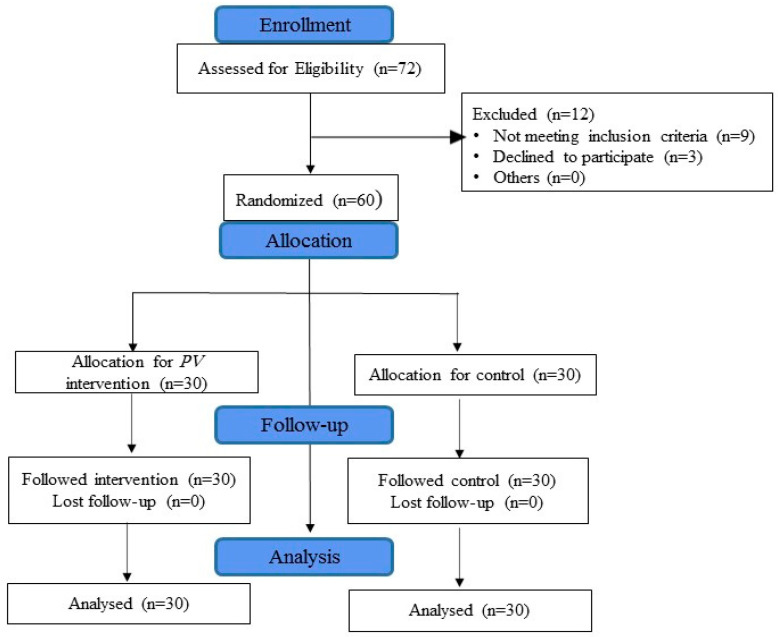
CONSORT flow diagram.

A total of 60 patients with kidney stones based on the inclusion criteria participated in the study, and none dropped out during the study. Figure 1 shows that the study subjects were randomly divided equally (30 in each group) into two groups as the intervention and control groups by using computer generated randomization allocation with the assistance of random allocation software.

#### Climate

The study was conducted during the summer in Al-Ahsa when the maximum temperature was 45 °C and the lowest was 29 °C. Both the PV group and control group were selected during the same period.

### 2.5. Data Collection Tools

A case record form was used to collect the data in this study. Initially the base line information was obtained before collecting the data. The tool included clinical symptoms, the anthropometric index, urinary biochemical parameters, and renal ultrasound scan findings.

#### 2.5.1. Sociodemographic Measures

The sociodemographic measures included age, gender, education, occupation, marital status, physical activity, diet pattern, presence of comorbidities, family history of kidney stones, and the quantity of drinking water per day. This baseline demographic information of both the PV group and control group was collected before the pre-test (Table 1).

#### 2.5.2. Clinical Assessment

The clinical symptoms were assessed by using a ‘yes’ or ‘no’ checklist, which included pain in the abdomen, pain in the lower back, pain in the urinary tract, a persistent urge to urinate, blood in the urine, feeling nauseated, vomiting, sweating, chills, and any other symptoms. The verbal rating scale [17] was used to assess the level of pain if the patient had pain. The participants were requested to mark the adjective that matched to the pain intensity [18]. In the verbal rating scale, there were two endpoints from ‘no pain at all’ to ‘extremely intense pain’. Between these two extreme points, different adjectives describing different pain-intensity levels were placed in the order of pain severity. The following five-point sets of descriptors were used to assess the pain [19]. They were ‘not at all’, ‘little bit’, ‘moderate’, ‘quite bit’, and ‘extreme’.

#### 2.5.3. Anthropometric Index

Measures of height and weight were assessed using an electronic scale. The body mass index (BMI) was calculated using the formula (mass (kg)/height (m)^2^) as per Quetelet’s index [20]. All assessments were done twice, and the average value was used in the analyses.

#### 2.5.4. Urinary Biochemical Parameters

The participants were asked to collect 24 h of urine to measure the urine volume. The calcium, magnesium, potassium, oxalate, uric acid contents, and urine power of hydrogen (pH) were tested in the laboratory during the pre and post intervention for both PV and control groups. The urinary pH measurement was performed using spontaneously voided urine. The PV group were instructed to do the post-test after 2 days of completing their intervention.

#### 2.5.5. Renal Ultrasound Scan

The renal ultrasound scan was performed for testing the following details during the pre- intervention and post-intervention periods. They were the location of the kidney stone, position of the stone, findings related to the size of calculi, number of calculi and other related findings.

### 2.6. Dietary Intervention

Green beans or PV were procured from the supermarket or grocery store. To prepare the PV extract, 250 g of fresh beans were taken, and washed thoroughly under clean running water. They were cut into small pieces for boiling. In a vessel, we added 1000 mL of water along with these cut beans and allowed it to boil. Then, we maintained the flame at medium and continued for 15 min. The water was filtered and allowed to cool and was kept in a container. This extract did not have either sugar or salt added. The participants were advised to drink on an empty stomach in the morning before breakfast at least one hour prior to eating anything. The prepared extract was to be used within 24 h of preparation. The patients of the interventional group were instructed to prepare the extract after the explanation and were asked to drink it thrice weekly (2.2 L to 2.5 L per week) for 6 weeks, probably every alternative day.

#### 2.6.1. Nutrients of PV

According to the United States Department of Agriculture (USDA) National Nutrient Database, approximately 150 g of beans yields 28 calories, 0.55 g of fat, 5.66 g of carbohydrates, 2.6 g of fibre contents, 1.94 g of sugar, and 1.42 g of protein. This amount also contains 17 mg of calcium, 1.2 mg of iron, 18 mg of magnesium, 30 mg of phosphorus, 130 mg of potassium, 24 µg of vitamin A, 52.5 µg of vitamin K and 32 µg of folate. Green beans are rich in polyphenols, which are a type of antioxidant that provides many health benefits [21].

#### 2.6.2. Compliance

During the initiation of the study, 4500 g of fresh beans in cartons containing 18 small packs (one pack had 250 g beans and there were three packs for each week) with clear labelled instructions were given to the study subjects in the intervention group. Each week, a total of 750 g (250 g into 3 days) beans were used to prepare the extract. The participants allocated to the PV group were verified over the telephone to ensure whether they had drunk their PV extract every week for six weeks. The percentage of compliance was calculated for each participant using the information provided. We considered it adequate adherence when >90% of the prescribed PV extract was consumed.

### 2.7. Outcome Measures

The outcome measures in this study were changes of urinary parameters and changes in the renal ultrasound findings. The urinary biochemical parameters were assessed by the 24 h urine as well as spontaneously voided urine. The clinical symptoms related to the renal calculi were assessed verbally. The BMI was assessed from measuring the weight and height. A questionnaire was used to collect the data of the participant’s demographic characteristics.

### 2.8. Data Analysis

The statistical analysis was performed using SPSS version 25.0 software (SPSS, Inc., Chicago, IL, USA). Statistical significance level was set at *p* < 0.05. The results are described as the mean ± standard deviation (SD) for continuous variables. The demographic characteristics of the subjects were compared between the interventional group and control group using an independent *t*-test for the quantitative variables and the Chi-square test for categorical variables. The paired t-test was used to compare the pre-intervention and post-intervention results within the groups. A one-way analysis of variance was used to identify the significant differences between the pre-intervention and post-intervention values of each group.

## 3. Results

Among the 60 patients (30 in each intervention and control group), 5 patients experienced pain in the abdomen (2 in the PV and 3 in the control groups), 7 patients experienced pain in the lower back (4 in the PV and 3 in the control groups), 5 patients experienced pain in the urinary tract (3 in the PV and 2 in the control groups).

From all participants, 8 patients reported feeling nauseated (5 in PV and 3 in the control groups), 11 patients had sweating (5 in the PV and 6 in the control groups), 6 patients had chills (3 in each PV and control group) and 3 patients reported that they had fever (1 in the PV group and 2 in the control group) during the pre-test assessment (Table 2).

Regarding the anthropometric index (Table 3) out of 60 patients (30 in PV and 30 in control group), the mean score of weight with SD is 71.82 kg ± 7.35 and 70.77 kg ± 7.87 in the pre-test and 71.59 kg ± 7.24 and 70.98 kg ± 7.97 in the post-test. Similarly, the mean score of height with SD was 164.9 cm ± 5.78 and 164.2 cm ± 5.95. There were no differences in height mean and SD value in pre and post- test in both the groups. There was a significant difference (*p*  <  0.05) observed in the mean score of BMI with SD in PV group 26.44 ± 2.7 and 26.36 ± 2.65. There was non-significance difference (*p* = 0.17716) in the mean score of BMI with SD in the control group at 26.28 ± 2.84 and 26.36 ± 2.88.

There were significant changes evidenced in the urine volume (*p* = 0.005), calcium level (*p* = 0.000), oxalate level (*p* = 0.000) and uric acid level (*p* = 0.000) statistically in the intervention group after PV. However, the magnesium (*p* = 14.5385) and urine pH levels did not show significant difference (Table 4)

**Figure 2:**
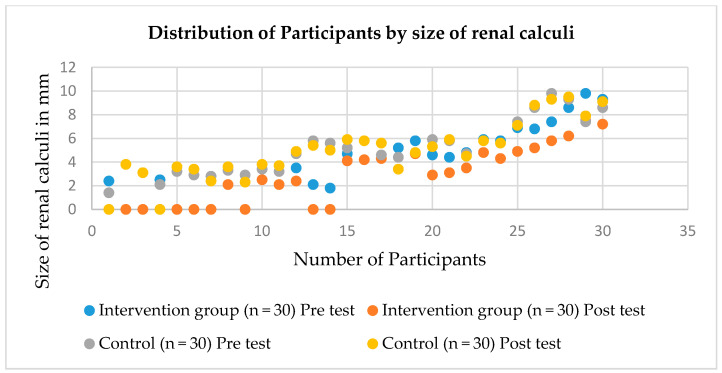
Distribution of renal Calculi.

A renal ultrasound scan was done before and after the intervention. The findings (Table 5) showed that the size of renal calculi is reduced in post-test more than in pre-test in the PV group than the control group. The mean score and SD of calculi is 4.74 ± 2.15 and 2.84 ± 2.41 in pre- and post-test, respectively, among PV group. There is statistically significant reduction of renal calculi size (*p* < 0.05). The number of calculi reduced after PV, which is evidenced (Figure 2).

## 4. Discussion

The kidney stones problem is found worldwide and is in especially high prevalence in hot climate areas like Saudi Arabia. [22]. Research has proved the historical evidence of the influence of diet on stone formation [23]. PV is a bean plant found in many countries and its health benefits are evidenced by literature. The researchers investigated to determine the effect of PV on urinary parameters in patients with kidney stones in this present study.

### 4.1. Effect on Symptoms of Kidney Stones

The analgesic effect of PV was proven by a study in which a high amount of polyunsaturated fatty acids (71.1 g/100 g), such as linolenic acid, found to be present in kidney beans, and linolenic acid decreases the level of prostaglandins and leukotrienes [24]. In our study, the pain intensity was reduced after the PV therapy from three to zero. There was a reduction in the number and percentage of clinical symptoms reported by the study participants.

### 4.2. Effect on BMI

There are studies that have proved the direct lipolytic effect on the mature adipocytes 3 T3-L1, from green bean extract concentrations with the consequent release of glycerol, and, due to that, the cell viability was not affected. The use of the green bean extract showed the antiadipogenic effects from the beginning of preadipocyte differentiation and reduced the lipid accumulation at the end of the process [25,26]. In this current study, the mean score of weight with SD was 71.82 kg ± 7.35 and 70.77 kg ± 7.87 in the pre-test, and 71.59 kg ± 7.24 and 70.98 kg ± 7.97 in post-test for the PV and control groups, respectively. There was a significant difference (*p*  <  0.05) observed in the mean score of the BMI with SD in the PV group at 26.44 ± 2.7 and 26.36 ± 2.65. These antiadipogenic effects were proven by many other researchers [27,28].

### 4.3. Effect on Urinary Biochemicals Parameters

In the present study, there were significant changes evidenced in the urine volume from 1962 mL ± 152.8 to 2005 mL ± 148.8 (*p* = 0.005), which showed the increase in volume after the intervention. In another study, there were no significant changes observed in the urinary volume. The urine analysis was done for a 24-h period with the use of an infusion of the plant. The urine volume was close to the minimum recommended in the literature, which is 2 L/day. In another study, researchers showed that the average values of the urine volume were 1927 mL, 2029 mL, and 2015 mL daily for the baseline, intervention and washout periods, respectively. [29,30,31,32]. This diuretic effect also helps to washout the calculi and prevents new stone formation.

There were changes in the urinary biochemical parameters observed in an animal study that provided PV seed extract. Few studies reported changes in the urinary biochemical values, such as calcium, potassium, magnesium, and sodium when testing the medicinal effects of selected plants, like *Phyllanthus Niruri*, among patients with renal calculi [33]. The current study tested the urine for calcium, which demonstrated significant changes observed in the calcium level (*p* = 0.000), but there was no significant change observed in magnesium level. The sodium level was not tested.

There were significant changes evidenced in the oxalate level (*p* = 0.000) and uric acid level (*p* = 0.000) after PV in the intervention group. These changes may interfere with certain stages of the crystallization in urine, such as a reduction in the nucleation, growth and aggregation of calcium oxalate crystals.

According to a study on the evaluation of patients with metabolic alterations at the baseline, a significant normalization of urinary uric acid and oxalate values occurred in those with hyperuricosuria and hyperoxaluria, respectively, and urine pH in the sample urinalysis did not change throughout that study and remained at a mean value between 6.0 and 6.1 [33,34,35]. In this study, there was a slight increase in the mean score of the urine pH from 6.03 ± 0.27 to 6.59 ± 0.31.

### 4.4. Effect on Ultrasound Findings of Kidney

The study participants were selected based on kidney stone size. The renal ultrasound scan was performed before and after the intervention of this study. The findings showed that there were changes in the size and number of kidney stones that were reduced in the post-test compared with the pre-test. The study was conducted on the medicinal plant effect on renal calculi, and the evaluation of renal calculi proved that there was a reduction in the number of calculi, which might be due to some of the participants passing them due to the diuretic effect of PV. However, the ultrasound scan is not a golden standard for the investigation of calculi as they are less than 10 mm size [36].

In this study, there were no changes in the magnesium level in the control group. In this study, a blood test was not performed to evaluate the biochemical parameters and the citrate level was not assessed. Overall, clinical studies with more patients are needed to investigate the effect of PV in patients with kidney stones. The same study could be performed to assess other relevant parameters like the blood and metabolic values. The intervention could be extended for a longer period of duration to validate the PV effect in the future. PV is a common and natural vegetable food resource available in many countries and can reduce the cost and expenditure of the health care system and its associated issues. This PV therapy can be advised for the prevention of kidney stones among the general population from a public health point of view.

## 5. Conclusions

This study was conducted to determine the effect of PV on urinary biochemical parameters, among patients with kidney stones. Based on the results, PV increased the urinary excretion of magnesium and potassium among the experimental group (*p* < 0.05) when compared to the control group. Therefore, we suggested that PV is an effective and safe method to reduce kidney stones occurrences and help to eliminate renal calculi among patients.

## Figures and Tables

**Table 1:** Reporting baseline demographic characteristics of intervention and control group.

Demographic Characteristics		PV Group	Control Group	*p* Value
		(*n* = 30)	(*n* = 30)	
		N (%)	N (%)	
Age	20–30 years	3 (10)	4 (13.3)	*p* = 0.957
	31–40 years	8 (26.7)	7 (23.3)	
	41–50 years	11 (36.6)	10 (33.4)	
	51–60 years	8 (26.7)	9 (30)	
Gender	Male	18 (60)	21 (70)	*p* = 0.417
	Female	12 (40)	9 (30)	
Educational level	Primary school	7 (23.3)	12 (40)	*p* = 0.188
	High school and diploma	17 (56.7)	16 (53.3)	
	University	6 (20)	2 (6.7)	
Employment status	Employed	24 (80)	22 (73.3)	*p* = 0.542
	Unemployed	6 (20)	8 (26.7)	
Marital status	Unmarried	2 (6.7)	3 (10)	*p* = 0.640
	Married	28 (93.3)	27 (90)	
Physical activity	Sedentary	17 (56.7)	16 (53.3)	*p* = 0.795
	Moderate	13 (43.3)	14 (46.7)	
Diet pattern	Vegetarian	1 (3.3)	1 (3.3)	*p* = 0.472
	Non-vegetarian	29 (96.7)	29 (96.7)	
Presence of comorbidities	Diabetes Mellitus	3 (10%)	5 (16.7)	*p* = 0.653
	Hypertension	2 (6.7)	1 (3.3)	
	None	25 (83.3)	24 (80)	
Family history of kidney stone	Yes	4 (13.3)	2 (6.7)	*p* = 0.389
	No	26 (86.7)	28 (93.3)	
Quantity of drinking water in liters per day	1	21 (70)	19 (63.3)	*p* = 0.584
	2–3	9 (30)	11(36.7)	

N = Number; % = Percentage.

**Table 2:** Clinical Assessment.

Clinical Symptoms	PV Group (*n* = 30)				Control (*n* = 30)			
	Pre-Test		Post-Test		Pre-Test		Post-Test	
	Yes	No	Yes	No	Yes	No	Yes	No
	N (%)	N (%)	N (%)	N (%)	N (%)	N (%)	N (%)	N (%)
Pain in the abdomen	2 (6.7)	28 (93.3)	0 (0)	30 (100)	3 (10)	27 (90)	2 (6.7)	28 (93.3)
Pain in lower back	4 (13.3)	26 (86.7)	1 (3.3)	29 (96.7)	3 (10)	27 (90)	3 (10)	27 (90)
Pain in urinary tract	3 (10)	27 (90)	0 (0)	30 (100)	2 (6.7)	28 (93.3)	1 (3.3)	29 (96.7)
Feeling nauseated	5 (16.7)	25 (83.3)	1 (3.3)	29 (96.7)	3 (10)	27 (90)	3 (10)	27 (90)
Sweating	5 (16.7)	25 (83.3)	3 (10)	27 (90)	6 (20)	24 (80)	5 (16.7)	25 (83.3)
Chills	3 (10)	27 (90)	0 (0)	30 (100)	3 (10)	27 (90)	1 (3.3)	29 (96.7)
Fever	1 (3.3)	29 (96.7)	0 (0)	30 (100)	2 (6.7)	28 (93.3)	0 (0)	30 (100)

N = Number; % = Percentage.

**Table 3:** Anthropometric index.

Measurements	PV Group (*n* = 30)			Control (*n* = 30)		
	Pre-Test	Post-Test		Pre-Test	Post-Test	
	Mean (SD)	Mean (SD)	Paired ‘t’ Test	Mean (SD)	Mean (SD)	Paired ‘t’ Test
Weight (kg)	71.82 ± 7.35	71.59 ± 7.24	t = 2.7162	70.77 ± 7.87	70.98 ± 7.97	t = 1.4196
			*p* = 0.01101 *			*p* = 0.1664 NS
BMI	26.44 ± 2.7	26.36 ± 2.65	t = 2.75;	26.28 ± 2.84	26.36 ± 2.88	t = 1.38;
			*p* = 0.01017 *			*p* = 0.17716 NS

* Significant; NS-Not Significant.

**Table 4:** Urinary Biochemical Parameters.

Urinary Parameters	PV Group (n = 30)			Control (n = 30)		
	Pre-Test	Post-Test		Pre-Test	Post-Test	Paired ‘t’ Test
	Mean (SD)	Mean (SD)	Paired ‘t’ test	Mean (SD)	Mean (SD)	
Urine volume (mL)	1962 ± 152.8	2005 ± 148.8	t = 3.0328	1992 ± 134.2	1964.2 ± 122.9	t = 1.013
			*p* = 0.005 *			*p* = 0.3194 (NS)
Calcium (mg)	205.4 ± 11.99	198.4 ± 12.52	t = 5.6538	201 ± 11.45	201.7 ± 12.74	t = 1.0838
			*p* = 0.000 *			*p* = 0.2874 (NS)
Magnesium (mg)	70.69 ± 4.37	81.28 ± 4.73	t = 7.55	72.82 ± 6.62	71.13 ± 4.3	t = 1.796
			*p* = 14.5385 (NS)			*p* = 0.08293 (NS)
Potassium (mEq)	44.07 ± 3.66	52.15 ± 4.37	t = 7.42946	43.99 ± 4.96	45.34 ± 5.44	t = 1.00713
			*p* = 0.000 *			*p* = 0.03181 (NS)
Oxalate (mg)	37.12 ± 5.38	33.02 ± 5.71	t = 4.6643	38.3 ± 5.57	38.65 ± 5.51	t = 1.6353
			*p* = 0.000 *			*p* = 0.1128 (NS)
Uric acid mg/dL	6.88 ± 0.7	6.31 ± 0.58	t = 5.8204	6.51 ± 0.87	6.7 ± 0.8	t = 1.7278
			*p* = 0.000 *			*p* = 0.09466 (NS)
pH	6.03 ± 0.27	6.59 ± 0.31	t = 7.4993	6.01 ± 0.37	6.13 ± 0.37	t = 1.5137
			*p* = 2.889 (NS)			*p* = 0.1409(NS)

* Significant; NS-Not Significant.

**Table 5:** Urinary Findings.

Renal Scan Findings	PV Group (*n* = 30)		Control (*n* = 30)	
	Pre-Test	Post-Test	Pre-Test	Post-Test
	N (%)	N (%)	N (%)	N (%)
Location of the kidney stone				
Right	13 (43.3)	9 (30)	10 (33.3)	9 (30)
Left	11 (36.7)	6 (20)	17 (56.7)	16 (53.3)
Both	6 (20)	5 (16.7)	3 (10)	3 (10)
Size of calculi				
1–3.9 mm	14 (46.7)	8 (26.7)	11 (36.7)	10 (33.3)
4–6.9 mm	12 (40)	10 (33.3)	13 (43.3)	12 (40)
7–9.9 mm	4 (13.3)	2 (6.7)	6 (20)	6 (20)
Number of calculi				
Single	22 (73.3)	14 (46.7)	25 (83.3)	23 (76.7)
Multiple	8 (26.7)	6 (20)	5 (16.7)	5 (16.7)

N = Number; % = Percentage.

## Abbreviations

BMI	Body Mass Index
CaOx	calcium oxalate (CaOx)
COVID-19	Corona virus disease-19
NS	Non-Significant
pH	Power of Hydrogen
PV	*Phaseolus Vulgaris*
SD	Standard Deviation
SPSS	Statistical Package for the Social Sciences
USA	United States of America
USDA	United States Department of Agriculture
